# A predictive tool particularly designed for elderly myeloma patients presenting with spinal cord compression

**DOI:** 10.1186/s12885-016-2325-y

**Published:** 2016-04-25

**Authors:** Dirk Rades, Antonio Jose Conde-Moreno, Jon Cacicedo, Theo Veninga, Niklas Gebauer, Tobias Bartscht, Steven E. Schild

**Affiliations:** Department of Radiation Oncology, University of Lubeck, Ratzeburger Allee 160, D-23538 Lubeck, Germany; Department of Radiation Oncology, Consorcio Hospital Provincial de Castellón, Castellón, Spain; Department of Radiation Oncology, Cruces University Hospital, Barakaldo, Vizcaya Spain; Department of Radiotherapy, Dr. Bernard Verbeeten Institute, Tilburg, Netherlands; Department of Hematology & Oncology, University of Lubeck, Lubeck, Germany; Mayo Clinic, Scottsdale, AZ USA

**Keywords:** Myeloma, Elderly patients, Spinal cord compression, Radiotherapy, Overall survival, Predictive tool

## Abstract

**Background:**

This study was performed to design a predictive tool that allows the estimation of overall survival (OS) of elderly myeloma patients (aged ≥65 years) presenting with myeloma-induced spinal cord compression (SCC).

**Methods:**

One-hundred-and-sixteen patients irradiated for motor deficits of the legs due to myeloma-induced spinal cord compression were retrospectively evaluated. Ten characteristics were analyzed for OS including age, interval between myeloma diagnosis and radiotherapy, other osseous myeloma lesions, myeloma type, gender, time developing motor deficits, number of affected vertebrae, ECOG-PS, pre-radiotherapy ambulatory status, and fractionation regimen. Characteristics that achieved significance on multivariate analysis were included in the predictive tool. The score for each characteristic was obtained from the 1-year OS rate divided by 10. The sum of these scores represented the prognostic score for each patient.

**Results:**

On multivariate analysis, myeloma type (hazard ratio 3.31; 95 %-confidence interval 1.75–6.49; *p* < 0.001), ECOG-PS (HR 5.33; 95 %-CI 2.67–11.11; *p* < 0.001), ambulatory status (HR 2.71; 95 % CI 1.65–4.57; *p* < 0.001), and age (HR 1.95; 95 % CI 1.03–3.78; *p* = 0.040) were significantly associated with survival. Sum scores ranged from 18 to 32 points. Based on the sum scores, three prognostic groups were designed: 18–19, 21–28 and 29–32 points. The corresponding 1-year survival rates were 0, 43 and 96 %, respectively (*p* < 0.001).

**Conclusions:**

This new predictive tool has been specifically designed for elderly myeloma patients with SCC. It allows estimating the survival prognosis of this patient group and supports the treating physicians when looking for the optimal treatment approach for an individual patient.

## Background

Spinal cord compression (SCC) due to malignant disease is an emergency that occurs in 5–10 % of adult oncologic patients [1, 2]. Myeloma accounts for 10–15 % of these patients. Since myeloma is a very radiosensitive entity, these patients were not included in a randomized trial that compared radiotherapy alone to radiotherapy plus upfront neurosurgery [3]. Thus, radiotherapy alone is considered the standard treatment of SCC from myeloma. Several fractionation regimens are used for malignant SCC including single-fraction, short-course multi-fraction and longer-course multi-fraction programs [1, 2]. The selection of the fractionation program for an individual patient should ideally take into account the patient’s remaining life time. Longer-course programs, which result in better local control of SCC than single-fraction and short-course programs, are the preferred treatment for patients with a more favorable survival prognosis [4–6]. In patient with a very good prognosis, stereotactic body radiation therapy may also be considered [7, 8]. In contrast, patients with a poor prognosis should spend as little of their remaining life time attending oncologic treatments and are, therefore, better candidates for multi-fraction short-course or single-fraction radiotherapy. These considerations imply that it is important to judge a patient’s survival time as accurately as possible, which is facilitated with predictive tools.

Due to demographic changes and improved oncologic therapy, the number of elderly patients with malignant diseases is constantly increasing. These patients are different from younger patients with respect to immune function, organ function and co-morbidities and may not tolerate aggressive treatment. Therefore, it is important to create predictive tools particularly designed for elderly patients. The goal of the present study was the development of a tool for predicting the overall survival (OS) of elderly myeloma patients presenting with SCC.

## Methods

One-hundred-and-sixteen elderly patients aged ≥65 years who received radiotherapy alone for motor deficits of the legs due to myeloma-induced SCC were retrospectively evaluated for OS. SCC associated with neurologic deficits is defined as manifest or true SCC, whereas asymptomatic SCC diagnosed only by spinal imaging or SCC associated with pain but not with neurologic deficits should be named pending SCC [2]. The patients included in the present study represent those elderly patients presenting with true SCC. The study has been approved by the local ethics committee (University of Lubeck). Elderly was defined according to the world health organization (chronological age of 65 years accepted as cut-off age for elderly or older persons) [9]. Furthermore, Orimo et al. defined elderly as a chronological age of 65 years or older [10].

Radiotherapy was performed with 6–18 MeV photon beams from a linear accelerator. Treatment volumes generally included one normal vertebra above and below those vertebrae involved. Patients with vertebral fractures with bony impingement of the spinal cord or nerve roots were not included, because they were candidates for decompressive surgery. The following ten characteristics were analyzed for associations with OS: age at the start of radiotherapy (≤71 vs. ≥72 years; median age: 71 years), interval between myeloma diagnosis and radiotherapy (≤15 vs. >15 months), other osseous myeloma lesions (no vs. yes), myeloma type (IgG vs. others), gender, time of developing motor deficits prior to radiotherapy (≤14 vs. >14 days), number of vertebrae affected by SCC (1–2 vs. ≥3), ECOG-PS (1–2 vs. 3–4), ambulatory status prior to radiotherapy (ambulatory without aid vs. ambulatory with aid vs. not ambulatory), and fractionation regimen (short-course radiotherapy with 8Gyx1 or 4Gyx5 over 1 week vs. longer-course radiotherapy with 3Gyx10 over 2 weeks, 2.5Gyx14/15 over 3 weeks, or 2Gyx20 over 4 weeks). These potential prognostic factors were selected in accordance with previous studies and survival tools developed for patients with malignant SCC [5, 11, 12]. For the univariate analysis of OS, the Kaplan-Meier-method and the log-rank test were used [13]. The characteristic that showed a significant association with OS (*p* < 0.05) or at least a trend (*p* < 0.07) were additionally included in a multivariate analysis performed with the Cox proportional hazards model. Hazard ratios (HR) and 95 %-confidence intervals (95 %-CI) were related to unit risk ratios (per unit change in regressor, unit = 1 month). Those characteristics that achieved significance in the multivariate analysis (*p* < 0.05) were considered for the creation of the tool predicting survival. The score for each significant characteristic was obtained from the 1-year OS rate divided by 10. The sum of these scores represented the prognostic score for each patient.

## Results

**Fig. 1:**
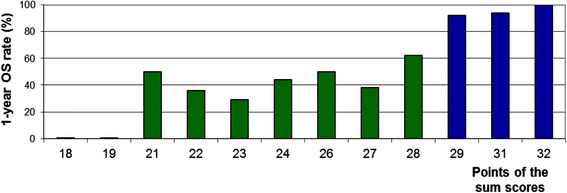
One-year overall survival rates of the sum scores

**Fig. 2:**
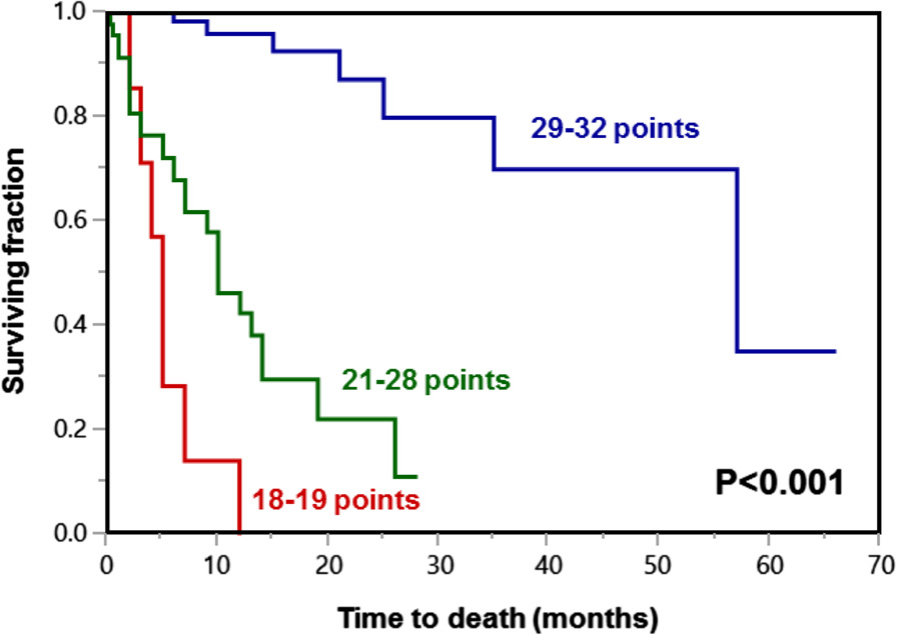
Kaplan-Meier curves for the overall survival of the three prognostic groups 18–19 points, 21–28 points and 29–32 points

On univariate analysis, OS was significantly influenced by myeloma type (*p* < 0.001), ECOG-PS (*p* < 0.001), and pre-radiotherapy ambulatory status (*p* < 0.001). In addition, age at radiotherapy showed a trend (*p* = 0.069). The 1-year OS rates of all investigated characteristics are summarized in Table 1. In the additional multivariate analysis, myeloma type (hazard ratio (HR) 3.31; 95 % confidence interval (CI) 1.75–6.49; *p* < 0.001), ECOG-PS (HR 5.33; 95 % CI 2.67–11.11; *p* < 0.001), pre-radiotherapy ambulatory status (HR 2.71; 95 % CI 1.65–4.57; *p* < 0.001), and age (HR 1.95; 95 % CI 1.03–3.78; *p* = 0.040) were significant and, therefore, included in the predictive tool (Table 2). The sum scores ranged from 18 to 32 points. The corresponding 1-year OS rates are shown in Fig. 1. Based on the sum scores, three prognostic groups were designed: 18–19 points (*n* = 7), 21–28 points (*n* = 47) and 29–32 points (*n* = 62). The corresponding 1-year survival rates were 0, 43 and 96 %, respectively (*p* < 0.001, Fig. 2). The intergroup comparisons revealed significant differences with respect to OS between groups 1 and 2 (*p* = 0.013) and between groups 2 and 3 (*p* < 0.001).

**Table 1:** Univariate analysis of overall survival

	Overall survival at 1 year (%)	*p*-value
Age		
≤71 years (*n* = 61)	74	
≥72 years (*n* = 55)	64	0.069
Interval myeloma diagnosis to radiotherapy		
≤15 months (*n* = 66)	76	
>15 months (*n* = 50)	59	0.15
Other osseous myeloma lesions		
No (*n* = 46)	75	
Yes (*n* = 70)	65	0.15
Myeloma type		
IgG (*n* = 72)	80	
Others (*n* = 44)	52	<0.001
Gender		
Female (*n* = 48)	62	
Male (*n* = 68)	75	0.15
Time developing motor deficits		
≤14 days (*n* = 50)	55	
>14 days (*n* = 66)	79	0.09
Number of vertebrae affected by SCC		
1–2 (*n* = 55)	69	
≥3 (*n* = 61)	69	0.54
ECOG-PS		
1–2 (*n* = 74)	85	
3–4 (*n* = 42)	36	<0.001
Ambulatory status prior to radiotherapy		
Ambulatory without aid (*n* = 37)	82	
Ambulatory with aid (*n* = 52)	77	
Not ambulatory (*n* = 27)	32	<0.001
Fractionation regimen		
Short-course radiotherapy (*n* = 39)	68	
Longer-course radiotherapy (*n* = 77)	69	0.88

**Table 2:** Characteristic significantly associated with overall survival in the Cox regression analysis and the corresponding scoring points based on the 1-year survival rates

	Scoring points
Age	
≤71 years	7
≥72 years	6
Myeloma type	
IgG	8
Others	5
ECOG-PS	
1–2	9
3–4	4
Ambulatory status prior to radiotherapy	
Ambulatory (with or without aid)	8
Not ambulatory	3

## Discussion

In oncology, individualization and personalization of the treatment taking into account a patient’s personal needs and expectations as well as his limitations due to a poor performance status or several co-morbidities has gained importance during recent years. The selection of an individual treatment approach is helped with an accurate picture of each patient’s expected survival time. In patients with a favorable prognosis, the risk of late treatment-related toxicities plays a more important role, whereas in patients with a poor prognosis, palliative aspects such as fast relief of symptoms and maintaining the maximum possible quality of life are more important. Therefore, the availability of predictive tools for estimating a patient’s survival prognosis is important to avoid over- or under-treatment. This is important in the palliative setting of SCC caused by a malignant disease. It has already been widely recognized that, because malignant diseases are quite different with respect to their biology and prognoses, separate predictive tools are needed for each entity, in order to provide the best treatment for an individual patient [2]. A survival score for patients irradiated for SCC from myeloma has already been developed three years ago [11]. In the multivariate analysis of that study, improved survival was significantly associated with better performance status (*p* = 0.036) and the ability to walk prior to radiotherapy (*p* = 0.037); absence of other osseous myeloma lesions showed a trend (*p* = 0.06). However, during recent years, oncologic research is increasingly focusing on elderly patients as a separate group who are generally more fragile. Thus, we decided to create a specific predictive tool for the group of elderly patients with SCC from myeloma. Although this is the first study reported so far that focuses on elderly patients irradiated for SCC from myeloma and, therefore, includes the largest series of these patients, the retrospective design should be kept in mind when interpreting the results and recommendations derived from these data. In retrospective studies, the risk of a hidden selection bias cannot be excluded. Furthermore, the score has not yet been validated. The relatively small number of 116 patients did not allow an appropriate validation of the present score at the moment. A validation needs to be performed in the future to demonstrate that the results of this study are sufficiently reliable. However, elderly patients with SCC from myeloma are relatively uncommon. Thus, validation of this scoring system may not be expected soon.

In the present study, of the ten characteristics that were analyzed for a potential association with OS, the four characteristics age, myeloma type, ECOG-PS and ambulatory function prior to radiotherapy were significant in the Cox proportional hazards model. In contrast, the fractionation regimen (short-course vs. longer-course radiotherapy) had no significant impact on OS in the current study. This finding agrees with the results of a previous study of SCC from myeloma including 218 patients of all age groups [11]. In contrast, a matched-pair study of patients with malignant SCC, which compared different longer-course radiotherapy programs (30Gy in 10 fractions to 37.5Gy in 15 fractions and 40Gy in 20 fractions), suggested a dose-effect relationship [14]. However, that study focused on patients with a very favorable survival prognosis. The 1-year OS rates were 79 % in the entire cohort, 78 % after 30Gy and 82 % after higher doses, respectively. Therefore, it is difficult to compare the findings of that matched-pair study to the results of the present study.

The four characteristics that were significant in the Cox proportional hazards model were considered in the predictive tool. Three different survival groups were designed (18–19 points, 21–28 points and 29–32 points) with 1-year OS probabilities of 0, 43 and 96 %, respectively. Patients with 18–19 points have the worst prognosis and may, therefore, be considered for a short-course radiotherapy program such as 4Gyx5 in 1 week, which has a similar effect on motor function but is less burdensome for the patients. Patients with 21–28 points have an intermediate survival prognosis and appear suitable for 3Gyx10 in 2 weeks, the world wide most commonly used fractionation regimen. Patients with 29–32 points have a much more favorable survival prognosis. According to a retrospective matched-pair analysis of 382 patients, longer-course programs with higher radiation doses such as 2.5Gyx15 (3 weeks) and 2Gyx20 (4 weeks) resulted in better local control of SCC and OS than 3Gyx10 (2 weeks) [14]. In that study, local control rates of SCC at 2 years were 71 % after 3Gyx10 and 92 % after higher doses (*p* = 0.012), and 2-year OS rates were 53 and 68 %, respectively (*p* = 0.032). Therefore, patients with 29–32 points should receive radiotherapy with total doses greater than 30Gy. Selected patients of this group may even be considered for stereotactic body radiation therapy, if the treatment complies with the tolerance doses of spinal cord and vertebral bone [15, 16].

## Conclusions

This new predictive tool contributes to the estimation of the survival prognosis of elderly myeloma patients presenting with SCC. Three prognostic groups were identified with significantly different 1-year OS probabilities. The tool facilitates the conception of personalized treatment for this group of patients requiring particular attention.

## Abbreviations

ECOG-PS: Eastern Cooperative Oncology Group performance score
Gy: gray
MeV: mega electron volts
SCC: spinal cord compression
